# Deficient Induction Response in a *Xenopus* Nucleocytoplasmic Hybrid

**DOI:** 10.1371/journal.pbio.1001197

**Published:** 2011-11-15

**Authors:** Patrick Narbonne, David E. Simpson, John B. Gurdon

**Affiliations:** 1 The Wellcome Trust/Cancer Research UK Gurdon Institute, The Henry Wellcome Building of Cancer and Developmental Biology, University of Cambridge, Cambridge, United Kingdom; 2 Department of Zoology, University of Cambridge, Cambridge, United Kingdom; National Cancer Institute, United States of America

## Abstract

Incompatibilities between the nucleus and the cytoplasm of sufficiently distant species result in developmental arrest of hybrid and nucleocytoplasmic hybrid (cybrid) embryos. Several hypotheses have been proposed to explain their lethality, including problems in embryonic genome activation (EGA) and/or nucleo-mitochondrial interactions. However, conclusive identification of the causes underlying developmental defects of cybrid embryos is still lacking. We show here that while over 80% of both *Xenopus laevis* and *Xenopus* (*Silurana*) *tropicalis* same-species androgenetic haploids develop to the swimming tadpole stage, the androgenetic cybrids formed by the combination of *X. laevis* egg cytoplasm and *X. tropicalis* sperm nucleus invariably fail to gastrulate properly and never reach the swimming tadpole stage. In spite of this arrest, these cybrids show quantitatively normal EGA and energy levels at the stage where their initial gastrulation defects are manifested. The nucleocytoplasmic incompatibility between these two species instead results from a combination of factors, including a reduced emission of induction signal from the vegetal half, a decreased sensitivity of animal cells to induction signals, and differences in a key embryonic protein (Xbra) concentration between the two species, together leading to inefficient induction and defective convergence-extension during gastrulation. Indeed, increased exposure to induction signals and/or *Xbra* signalling partially rescues the induction response in animal explants and whole cybrid embryos. Altogether, our study demonstrates that the egg cytoplasm of one species may not support the development promoted by the nucleus of another species, even if this nucleus does not interfere with the cytoplasmic/maternal functions of the egg, while the egg cytoplasm is also capable of activating the genome of that nucleus. Instead, our results provide evidence that inefficient signalling and differences in the concentrations of key proteins between species lead to developmental defects in cybrids. Finally, they show that the incompatibilities of cybrids can be corrected by appropriate treatments.

## Abstract 2

Defects in induction signaling and response underlie the nucleocytoplasmic incompatibility between two evolutionarily distant frog species, while specific treatments partially restore this response in explants and whole embryos.

## Author Summary

When two species evolve separately for several million years, their respective genomes accumulate many small changes that together are responsible for the differences in their characters. Some of these affect the way eggs are prepared inside the germline, and/or how embryos develop, such that the egg cytoplasm of a given species can only support development promoted by its own genome or nucleus. Thus, developmental incompatibility arises between the cytoplasm and the nucleus of distant species during evolution and we don't know its mechanism. We have studied this phenomenon in an advantageous system using two evolutionarily distant frog species (*Xenopus laevis* and *Xenopus tropicalis*). We found that hybrid frog embryos with *X. laevis* cytoplasm and *X. tropicalis* nuclei are always defective in an important process that is necessary to generate morphogenetic cell movements during development. Through a series of experiments in which we dissect out and/or recombine parts of such hybrid embryos and observe their behaviour in culture, we show that this phenomenon occurs because of malfunctions in the signalling cascade that is responsible for generating these cell movements. Thus, we postulate that inefficient molecular signalling contributes to the death of such hybrids.

## Introduction

Investigation of the mechanisms generating the characters or phenotypes during development has revealed the importance of the nucleus and its DNA content in directing developmental processes [1]–[4]. Nonetheless, the cytoplasm of the egg is responsible for the specification of many early aspects of development, including polarity, as well as cleavage type and developmental timing [3]–[6]. In principle, for the nucleus of one species to be compatible with the cytoplasm of the egg of another species, the foreign species' nucleus must not interfere beyond a certain threshold with the maternally regulated developmental processes of the cytoplasmic (egg) species, while the recipient egg cytoplasm also needs to fully “activate” and support development promoted by the foreign nucleus. Thus, as a general rule, it is possible to generate viable offspring via interspecies Somatic Cell Nuclear Transfer (iSCNT) only if the egg cytoplasm and the donor nucleus come from two very closely related species or from sub-species, which develop in a highly similar manner. Indeed, if the two species used are sufficiently distant, the resulting embryos rarely progress normally through embryonic development and often arrest at the stage of EGA or soon after [2],[3],[7],[8].

One of the first scientists who became interested in this field was Baltzer, who revealed the importance of the nucleus in development using androgenetic *Triturus* cybrids. Indeed, he observed that when a sperm from one species is combined with the egg cytoplasm of another species, androgenetic development differs from that when the sperm is of the same species as the egg, and in fact leads to severe developmental defects [1]. He, and others, further recorded differences between the development of reciprocal androgenetic cybrids [9]–[11], which could in principle suggest that the basis of the incompatibilities between the respective nuclei and cytoplasms of two given species might not necessarily be reciprocal. Later, the method of nuclear transfer [12] not only enabled the transplantation of diploid nuclei into enucleated eggs in virtually any species combinations, but also allowed the transfer of nuclei from cybrid embryos back to their own species egg cytoplasm. Using this technique, Moore (1958) showed that the nucleocytoplasmic incompatibilities between two *Rana* species (*R. pipiens* and *R. sylvatica*) led to irreversible nuclear damage [13]. Similar conclusions were later attained when back-transfer experiments were performed with the cybrids made from two *Xenopus* species (*X. laevis* and *X. tropicalis*), suggesting that irreversible nuclear damage may be a common effect of nucleocytoplasmic incompatibilities [14]. Interestingly, cybrid lethality was shown to occur even in a combination (*R. palustris* nuclei into *R. pipiens* cytoplasm) in which no cytologically detectable chromosome damage was found to occur and back-transferred embryos developed normally [15]–[17], suggesting that nuclear damage is not the whole explanation for developmental defects in cybrids. Also, a few experiments in which pieces of cybrid embryos were grafted onto normal embryos of either parental species suggested that the developmental defects of these embryos were cell autonomous, as contact with normal tissue did not rescue their developmental potentials [14],[16]. Finally, a more extreme cybrid combination (*D. pictus* nucleus into *X. laevis* egg cytoplasm) also generated by iSCNT, arrested before gastrulation, had reduced mRNA synthesis and did not initiate rRNA synthesis [18]. It, however, remains unclear whether these defects were the primary causes of the arrest, or secondary to other incompatibilities.

Work later performed in fish using iSCNT suggested that major differences in chromosome numbers could be one of the essential factors causing the nucleocytoplasmic incompatibilities [19],[20]. Interestingly, chromosome loss was observed in lethal fish hybrids generated by cross-fertilization, while in one such combination, phospho-histone H3 abnormally persisted on the lagging chromosomes during anaphase [21],[22]. Consistent with the amphibian work, cybrid lethality can, however, occur without any obvious defects in chromosome segregation, since a fish cybrid combination (goldfish nucleus into loach egg cytoplasm) that does not suffer from chromosome elimination is embryonic lethal [20],[23].

In the meantime, massive experimentation with iSCNT in mammals has also explored the limits of this technique and provided new insights regarding the potential causes of the developmental arrest in lethal cybrid combinations. One main conclusion derived from several reports suggested that a major barrier to cybrid development must be manifested at the stage of EGA, since it coincides with the stage of arrest of a majority of lethal mammalian cybrid combinations [7],[8]. This hypothesis was recently supported by transcriptional analyses [24]–[27]. A second possibility is a potential incompatibility between the maternal mitochondrial genome and that of the foreign species nucleus, leading to defects in mitochondrial function in cybrids [7]. A reason for this suspicion comes from the fact that higher mutation rates (compared to that of genomic DNA) combined with maternal inheritance can lead to rapid divergence in mitochondrial DNA during evolution [28]. Also, the efficiency of same-species bovine SCNT is increased if the donor and recipient cells have the same mitochondrial haplotype [29], while ATP levels were reduced in chimpanzee/bovine iSCNT embryos [27]. Finally, evolutionary distances as little as 8–18 million years lead to fatal defects in oxidative phosphorylation in primate and rodent xenomitochondrial cybrid cell lines [30],.

Pioneering work has thus established that there are developmental incompatibilities between the nucleus and the cytoplasm of sufficiently distant species. Yet the rules that determine the compatibility between the maternal cytoplasmic content and that of the nucleus in the context of early development remain poorly defined. Equally obscure are the precise initial faults in the developmental mechanisms that eventually lead to the arrest in cybrid embryos. To better understand the nature of the nucleocytoplasmic incompatibilities that exist between relatively distant species, we have analysed here the developmental potentials and defects of reciprocal *X. laevis* and *X. tropicalis* hybrids and those of the lethal cybrids formed by the combination of a haploid *X. tropicalis* sperm nucleus and a *X. laevis* egg cytoplasm.

## Results

In order to abbreviate the designation of the many kinds of embryos that are referred to in this article, the following convention has been adopted. A first italicized letter refers to the species origin of the egg, followed by an “x,” standing for “fertilized with” or “cross-fertilized with,” and a second italicized letter, representing the sperm species. The letters “*l*” and “*t*,” respectively, represent *X. laevis* and *X. tropicalis*. If a component's nucleus has been inactivated, using ultraviolet (UV) irradiation [32],[33], square brackets surround the relevant letter. Thus, using this convention, “[*l*]x*l*” means an embryo resulting from a UV-irradiated *X. laevis* egg fertilized with *X. laevis* sperm.

When considering the study of potential incompatibilities between the nucleus of one species and the egg cytoplasm of another, it is first important to determine whether there is something within that foreign nucleus that could interfere with the maternal functions of the egg cytoplasm. One way to address this question consists of comparing the development of haploid embryos with that of hybrids, in which one copy of a foreign species (sperm) genome is added (with very little cytoplasm) to the egg of another species. In the first place, we therefore asked how well *X. laevis* and *X. tropicalis* haploid embryos develop, and in the second, we compared the development of haploids of each species with that of the reciprocal hybrids.

### Androgenetic Haploid Development Gives Rise to Swimming Tadpoles in Both *X. laevis* and *X. tropicalis*

**Figure 1:**
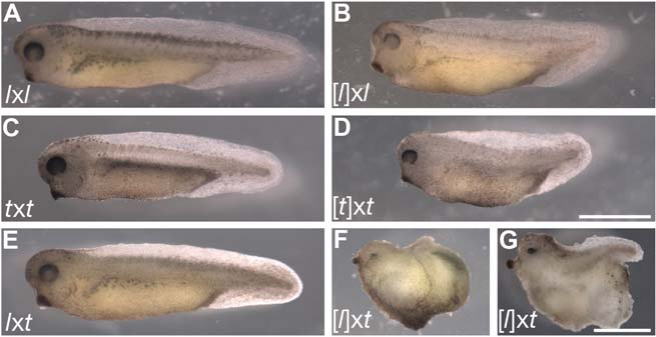
Early development of *Xenopus* androgenetic haploids, hybrids, and cybrids. (A–D) Haploid development frequently gives rise to stunted swimming tadpoles in both *X. laevis* and *X. tropicalis*. Typical stage 38 (A) *l*x*l*, (B) [*l*]x*l* (*l*x[*l*] were identical (unpublished data)), (C) *t*x*t*, and (D) [*t*]x*t* embryos are shown. (E) Haploid *X. laevis* development is improved by the addition of a *X. tropicalis* sperm nucleus. A typical stage 38 *l*x*t* hybrid is shown. (F–G) [*l*]x*t* cybrids have a reduced developmental capacity compared to [*l*]x*l or* [*t*]x*t* and never form swimming tadpoles. Two of the most developmentally advanced [*l*]x*t* cybrid postneurulae after ∼72 h at 23°C are shown. Substantial posterior axis elongation such as in the individual shown in (G) is very rare and occurs in less than 1% of [*l*]x*t* cybrids. Scale bars in (A–B, E–G) and (C–D): 1 mm.

In all haploid Anura, the onset of gastrulation is delayed by the time it takes for all the cells to undergo approximately one additional division, until they reach the same nucleocytoplasmic volume ratio as in diploid embryos [34]. In addition to this developmental retardation, *X. laevis* haploid embryos are microcephalic, have a shorter axis, and suffer from lordosis and a bulging abdomen (Figure 1A–B; [35]). Haploids also have a feeble heart, are much less active than diploids, and are subject to oedema, such that no haploid *X. laevis* has ever reached metamorphosis [34],[35]. Consistent with this, [*l*]x*l* embryos showed all of the early phenotypes described above (Figure 1A–B, Table 1, Videos S1–S2). Development of *l*x[*l*] embryos was also briefly investigated and found to be identical to that of [*l*]x*l* embryos (unpublished data). We further found that the development of [*t*]x*t* embryos was comparable to that of [*l*]x*l* or *l*x[*l*] in all respects (Figure 1C–D, Table 1). Therefore, both species possess a roughly equal early developmental potential in the androgenetic haploid state, developing into similarly advanced stunted swimming tadpoles with a frequency above 80% (Figure 1A–D, Table 1).

**Table 1:** Success of embryonic development in *Xenopus* haploids, hybrids, and cybrids.

Embryo	Normal Stage 2† (n)	Regular Stage 9 (%)	Died Between Stage 9–13 (%)	Died Between Stage 13–19 (%)	Normal Stage 19 (%)	Died as an Abnormal Postneurulae (%)	Swimming Tadpole
							Stunted (%)	Normal (%)
*l*x*l*	122 (4)	122 (100)	0 (0)	0 (0)	120 (98)	0 (0)	3 (2)	119 (98)
*l*x*t*	147 (4)	144 (98)	3 (2)	0 (0)	140 (95)	0 (0)	5 (3)	139 (95)
[*l*]x*l*	79 (5)	78 (99)	0 (0)	0 (0)	76 (96)	2 (3)	75 (95)	2 (3)
[*l*]x*t*	101 (8)	91 (90)	28 (28)	33 (33)	0 (0)	40 (40)	0 (0)	0 (0)
*t*x*l* ‡	41 (4)	40 (98)	41 (100)	0 (0)	0 (0)	0 (0)	0 (0)	0 (0)
*t*x*t*	83 (2)	82 (99)	0 (0)	0 (0)	81 (98)	1 (1)	3 (4)	79 (95)
[*t*]x*l* ‡	0 (3)	—	—	—	—	—	—	—
[*t*]x*t*	106 (7)	101 (95)	6 (6)	2 (2)	83 (78)	11 (10)	87 (82)	0 (0)

n, number of different male-female combinations from which the results were added.

† , unfertilized eggs and embryos that showed an abnormal or incomplete first cleavage were excluded from this analysis.

‡ , cross-fertilization efficiency was very low (∼3% in the best cases) for *X. laevis* sperm on *X. tropicalis* eggs; our attempts at cross-fertilizing UVed *X. tropicalis* eggs with *X. laevis* sperm were unsuccessful.

### Distinct Development of Reciprocal *X. tropicalis* and *X. laevis* Hybrids

We then asked whether the addition of a haploid nucleus from one of these species would interfere with haploid development of the other species. Reciprocal in vitro cross-fertilization between *X. laevis* and *X. tropicalis* has previously been reported [36]–[38]. Whereas the cross-fertilization of the eggs of *X. tropicalis* with *X. laevis* sperm is very inefficient (∼3%), that of *X. laevis* eggs with *X. tropicalis* sperm is comparable to that of *X. laevis* self-fertilization (unpublished data; [37]). It has been stated that *l*x*t* hybrids are viable and can develop to the adult stage [36], yet further characterization was lacking. We have thus analysed the development of the reciprocal hybrids that can be generated from *X. laevis* and *X. tropicalis*.

The *l*x*t* hybrid embryos develop into swimming tadpoles (stage 40) with a frequency that is comparable to that of *l*x*l* or *t*x*t* control embryos (Figure 1E, Table 1, Text S1, Figure S1), suggesting that the addition of a haploid set of *X. tropicalis* chromosomes does not interfere with gynogenetic haploid *X. laevis* development. Interestingly, the defects of *l*x*t* hybrids are highly reminiscent of those seen in *X. laevis* haploids ([*l*]x*l* or *l*x[*l*]), although they have a markedly decreased severity in the hybrids (Figure 1A–B,E, Table 1). Thus, an additional haploid set of *X. tropicalis* chromosomes is in fact beneficial to *X. laevis* haploid development since *l*x*t* hybrids develop further than *X. laevis* haploids ([*l*]x*l* or *l*x[*l*]), which never reach metamorphosis (Figures 1A–E, S1, Table 1; [34],[35]).

The reciprocal *t*x*l* hybrid embryos all died as late blastulae or very early gastrulae (Table 1). This indicates that an additional haploid set of *X. laevis* chromosomes is very damaging to the early development of a gynogenetic *X. tropicalis* haploid. We have not further characterized this arrest. The results thus suggest that a haploid *X. tropicalis* nucleus does not interfere with *X. laevis* maternal and embryonic development, while a haploid *X. laevis* nucleus severely interferes with *X. tropicalis* maternal or embryonic developmental processes.

### Nucleocytoplasmic Incompatibilities between *X. tropicalis* Nuclei and *X. laevis* Egg Cytoplasm Lead to Early Gastrulation Defects

Since a haploid *X. tropicalis* nucleus did not interfere with *X. laevis* haploid development, we next asked whether the cytoplasm of a *X. laevis* egg can support the normal development promoted by a *X. tropicalis* nucleus. Earlier experimentation with iSCNT suggested that the cytoplasm of the *X. laevis* egg was not capable of reprogramming and/or sustaining normal development promoted by a *X. tropicalis* neurula/tailbud stage somatic nucleus [14]. If this nucleocytoplasmic incompatibility between the *X. laevis* egg cytoplasm and a *X. tropicalis* somatic nucleus was not due to defects linked to nuclear transfer or reprogramming, the cytoplasm of a *X. laevis* egg should also be incapable of sustaining the normal development promoted by a *X. tropicalis* sperm nucleus to the swimming tadpole stage. We therefore compared the development of [*l*]x*t* cybrids to that of [*l*]x*l* or [*t*]x*t* same-species controls. The [*l*]x*t* cybrids developed relatively normally at first and were indistinguishable from control [*l*]x*l* androgenetic haploids until they reached the beginning of gastrulation and the appearance of the dorsal lip of the blastopore (stage 10.25), approximately 1 h after *l*x*l* diploids (Table 1, Videos S1–S2). However, the [*l*]x*t* cybrid embryos subsequently showed developmental retardation and consistently failed to close their blastopore, formed abnormal neurulae, and all died as abnormal, non-swimming postneurulae (Figure 1F–G, Table 1, Video S2). The proportion of embryos reaching a postneurula stage ranged from <10% to >80% depending on male/female combinations (or egg batches). The most developmentally advanced of these cybrid embryos had a rudimentary sucker, microcephalic head, and pigmented elementary eyes. A few (<5%) also sporadically underwent bursts of rhythmic muscular contractions and/or developed a primitive caudal fin, and very few (<1%) also showed posterior axis elongation (Figure 1G shows the most developed [*l*]x*t* embryo obtained, while Figure 1F shows a more typical example). Exceptionally well-developed [*l*]x*t* cybrid individuals could survive for up to almost a week. Overall, the terminal phenotype of these [*l*]x*t* cybrid embryos is very similar to those (diploids) previously obtained by iSCNT [14]. One difference, however, is that they are less elongated, which is likely to be the result of the difference in ploidy, since same-species *Xenopus* haploids are readily characterized by reduced axis elongation (Figure 1A–D; [34],[35]). Thus, [*l*]x*t* androgenetic haploid cybrids have a reduced developmental potential compared to same-species androgenetic haploid controls ([*l*]x*l* or [*t*]x*t*), demonstrating the existence of a developmental nucleocytoplasmic incompatibility between these two species that is not due to nuclear transfer or reprogramming defects. Even though the replication of *X. tropicalis* nuclei in *X. laevis* cytoplasm may trigger unknown nuclear aberrations [14], chromosome loss was not observed in the [*l*]x*t* cybrid embryos (4/4 [*l*]x*t* embryos had cells in which the expected haploid chromosome complement of *X. tropicalis* was clearly visible in metaphase squash preparations; unpublished data).

Attempts to generate the reciprocal [*t*]x*l* androgenetic haploid cybrid were unsuccessful, probably owing to the low efficiency of cross-fertilization in this direction (Table 1), and thus the developmental potential of a haploid *X. laevis* nucleus in a *X. tropicalis* egg cytoplasm remains undefined. These results thus indicate that even though the presence of a *X. tropicalis* haploid nucleus does not interfere with (and even improves) gynogenetic *X. laevis* development, the *X. laevis* egg cytoplasm does not support the development that is promoted by a *X. tropicalis* nucleus as well as the *X. tropicalis* egg cytoplasm. Furthermore, it establishes the onset of gastrulation (stage 10) as the critical stage where the nucleocytoplasmic incompatibility is first manifested.

### Gastrulation Defects of Cybrids Are Not Due to Major Defects in EGA or Protein Synthesis

**Figure 2:**
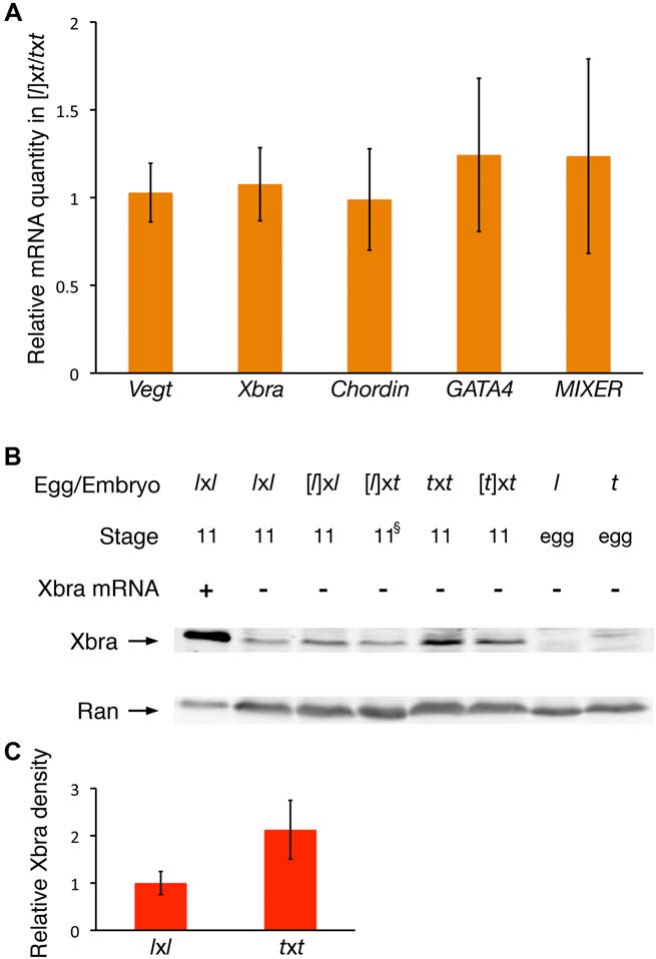
EGA and translation appear normal in cybrid embryos. (A) Transcription of key embryonic genes is normal in [*l*]x*t* cybrids. Real-time RT-PCRs were carried out to compare relative mRNA/total RNA quantities for the indicated genes in stage 10.25 [*l*]x*t* cybrid embryos as compared to that in *t*x*t*. Relative mRNA quantities were found to be identical for *Vegt* and *Xbra* at stage 10.25 between *t*x*t* and [*t*]x*t* (unpublished data). Error bars represent the standard deviation between three to six replicate experiments, each of which is the average of three to six single embryos. No significant difference was found (*p*>0.05; two-tailed *t* test) for any of the genes tested. (B) Xbra protein is synthesized normally from the *X. tropicalis* genome by the *X. laevis* cytoplasm. Western blot analysis was carried out to evaluate Xbra protein levels in stage 11 embryos of the indicated combinations. ^§^ [*l*]x*t* embryos never morphologically reach stage 11, but they were harvested when [*l*]x*l* siblings reached stage 11. Ran was used as a loading control as it is present at the same concentration in the eggs of both species [67]. (C) Quantification of pixel intensity indicates a significant (∼2-fold) increase in Xbra protein concentration at stage 11 in *t*x*t* relative to *l*x*l* (*p* = 0.014; one-tailed *t* test with unequal variance; *n* = 4).

A major barrier to the development of cybrid embryos is believed to reside at the stage of EGA [7],[18],[24]–[27]. Suppression of transcription with α-amanitin (intra-cytoplasmic concentration of 50 µg/ml) causes *X. laevis* embryos to arrest prior to gastrulation (unpublished data; [39]). It is therefore plausible that the components of the *X. laevis* egg cytoplasm are unable to efficiently activate transcription from the *X. tropicalis* genome, resulting in the observed gastrulation defects. To test this, we used quantitative RT-PCR to evaluate the mRNA content of several embryonically transcribed genes in stage 10.25 [*l*]x*t* cybrid embryos. The relative quantity of transcripts for *Xbra*, *Chordin*, *Gata4*, and *Mixer* at this stage in [*l*]x*t* cybrids was not significantly different from *t*x*t* or [*t*]x*t* control embryos (Figure 2A). We therefore conclude that the gastrulation defects of [*l*]x*t* cybrids do not arise from a generalized inefficient EGA.

To exclude the possibility that differences in the splicing or translation machineries between the two species could instead lead to inefficient protein synthesis from these properly concentrated embryonic transcripts following EGA, we investigated the production of Xbra protein in [*l*]x*t* cybrids. Western blot comparison of stage 11 embryos revealed that the relative concentration of Xbra protein in [*l*]x*t* cybrid embryos was similar to that in control *X. laevis* embryos (*l*x*l* and [*l*]x*l*) (Figure 2B). It may be important to note that Xbra protein concentration is markedly reduced in *X. laevis* egg-based embryos (*l*x*l*, [*l*]x*l*, [*l*]x*t*) relative to *X. tropicalis* egg-based embryos (*t*x*t*, [*t*]x*t*) (Figure 2B–C). This suggests that the concentration of Xbra protein at stage 11 is different in *X. laevis* and *X. tropicalis* embryos, and maternally/cytoplasmically regulated in the cybrid. However, since the level of Xbra protein present in the *X. laevis* egg-based embryos is similar regardless of whether it is encoded by a *X. laevis* or *X. tropicalis* genome, these results suggest that the early gastrulation defects of the cybrid embryos do not result from a generalized deficiency in EGA or protein synthesis.

The last phase of EGA in *Xenopus* consists of the activation of rDNA transcription and nucleologenesis [40],[41]. Nucleologenesis requires factors present in the oocyte nucleolus in mammalian embryos [42], and was defective in cybrids generated by iSCNT [18],[26],[43]. To verify whether the last phase of EGA is completed in [*l*]x*t* cybrids, we analysed nucleologenesis (the nucleolus itself results from active rDNA transcription [44]) in these embryos. No statistical difference was, however, found regarding nucleoli numbers between the nuclei of the [*l*]x*t* cybrids and control haploids ([*l*]x*l* or [*t*]x*t*) (Figure S2A–G), indicating that the *X. laevis* cytoplasm efficiently recognizes the *X. tropicalis* nucleolar organizer. Nucleolar integrity in [*l*]x*t* cybrids was further confirmed by the broad intra-nucleolar distribution of fibrillarin [26], identical to the controls (Figure S2H–J). Interestingly, *l*x*t* hybrid embryos had significantly fewer nuclei with two nucleoli than either diploid controls (*l*x*l* and *t*x*t*) (Figure S2A–G), suggesting that one of the nucleolar organizers is partially dominant. Our results, however, suggest that nucleologenesis, and thereby rRNA synthesis, is successful and that EGA is therefore completed in [*l*]x*t* cybrids; their early gastrulation defects must therefore arise from other incompatibilities.

### Gastrulation Defects of Cybrids Are Not Due to Energy Stress

**Figure 3:**
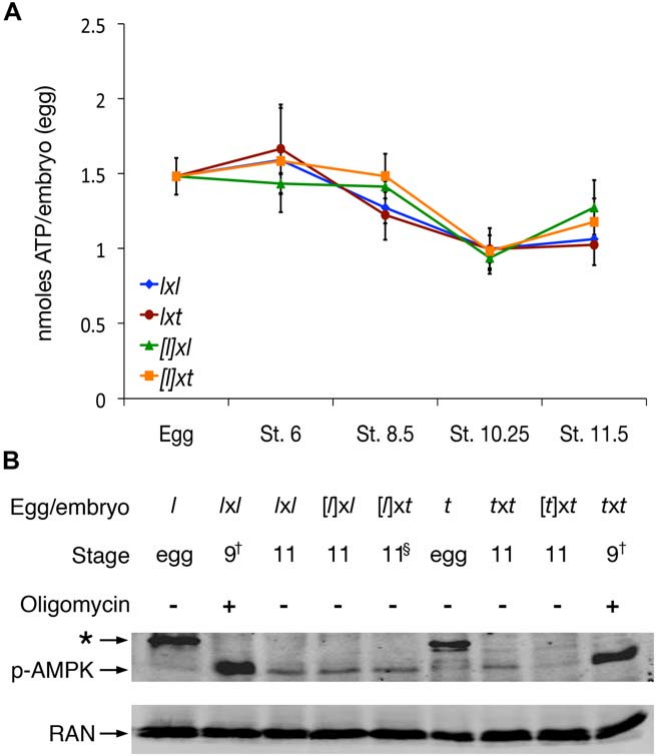
Cybrid early gastrulae are not energy-deficient. (A) ATP content, measured using a luciferase-based assay, is normal in cybrid embryos. Samples were collected every 3 h, except for the interval between stage 8.5 and 10.25 for [*l*]x*l* and [*l*]x*t*, which was about 4 h. Each point on the graph represents the average measurement (triplicate readings) of two pools of five embryos, all from the same *X. laevis* female. Error bars represent the standard deviation between the average readings of the two pools. (B) The activity of a key energy stress-sensing kinase is normal in [*l*]x*t* cybrids. Activated AMPK was detected on a Western blot using monoclonal anti-phospho-AMPK antibodies. Oligomycin, an inhibitor of mitochondrial ATP synthesis [46], was used as a positive control to induce energy stress. Ran was used as a loading control [67]. All *X. laevis* egg-based embryos are from the same female; *X. tropicalis* egg-based embryos are from two different females, which might explain the slight difference in AMPK phosphorylation observed between the two samples. * Non-specific band present in the eggs of both *X. laevis* and *X. tropicalis*. ^†^ Oligomycin-treated embryos (40 µM) arrested development at stage 9, but were harvested when diploid controls reached stage 11. ^§^ [*l*]x*t* embryos morphologically never reach stage 11, but they were harvested when their [*l*]x*l* control siblings reached stage 11.

An incompatibility between the maternal species mitochondrial genome and the foreign species nuclear-encoded mitochondrial genes could lead to deficient energy production and underlie lethality in cybrids [7],[45]. Mitochondrial ATP synthesis is indeed required for *Xenopus* embryos to initiate gastrulation (100% of *X. laevis* or *X. tropicalis* embryos (*n* = 30 each) arrested at stage 9 when cultured in 40 µM oligomycin (unpublished data), an inhibitor of mitochondrial ATP synthase [46]). We used a luciferase-based assay to determine the absolute ATP content at various time points during early embryonic development in the diverse kinds of *X. laevis* egg-based embryos (*l*x*l*, [*l*]x*l*, *l*x*t*, and [*l*]x*t*). The average number of ATP molecules per *X. laevis* egg obtained by this method was 1.5 nmoles (from three different frogs), close to the 1.6 nmoles for in vitro matured oocytes that was previously measured using chromatography [47]. Overall, the ATP content in all *X. laevis* egg-based embryos tested decreased until stage 10.25 to about 2/3 of the egg content, and then remained constant or slightly increased until stage 11.5 (Figure 3A). The ATP content curves of the two kinds of diploid embryos (*l*x*l* and *l*x*t*) were very similar to each other, while those of the two kinds of haploid embryos ([*l*]x*l* and [*l*]x*t*) appeared slightly different from the diploid curves, which may reflect different energy dynamics between haploid and diploid embryos. No statistical difference (*p*>0.05; two-tailed *t* test) in ATP content was found between [*l*]x*t* cybrids and control [*l*]x*l* sibling embryos at any time point until stage 11.5 (Figure 3A). Thus, we conclude that the early gastrulation defects in [*l*]x*t* cybrid embryos are not due to reduced ATP levels.

Energy stress in all eukaryotes is detected in a very sensitive manner by the AMP-activated protein kinase (AMPK), which becomes phosphorylated in its activation loop following an increase in the AMP∶ATP ratio [48]. We have thus used anti-phospho-AMPK antibodies to detect AMPK phosphorylation in various kinds of embryos at stage 11, well after the onset of the gastrulation defects of [*l*]x*t* cybrids. The level of AMPK phosphorylation in these cybrids was similar to that of control embryos (Figure 3B). Therefore, we conclude that the gastrulation defects in [*l*]x*t* cybrid embryos are not due to ATP depletion or energy stress. It appears unlikely that an incompatibility between the *X. laevis* mitochondria and the *X. tropicalis* nucleus that would not affect ATP levels could explain the early gastrulation defects occurring in these cybrids.

### Poor Convergence-Extension in Gastrulating Cybrids

**Figure 4:**
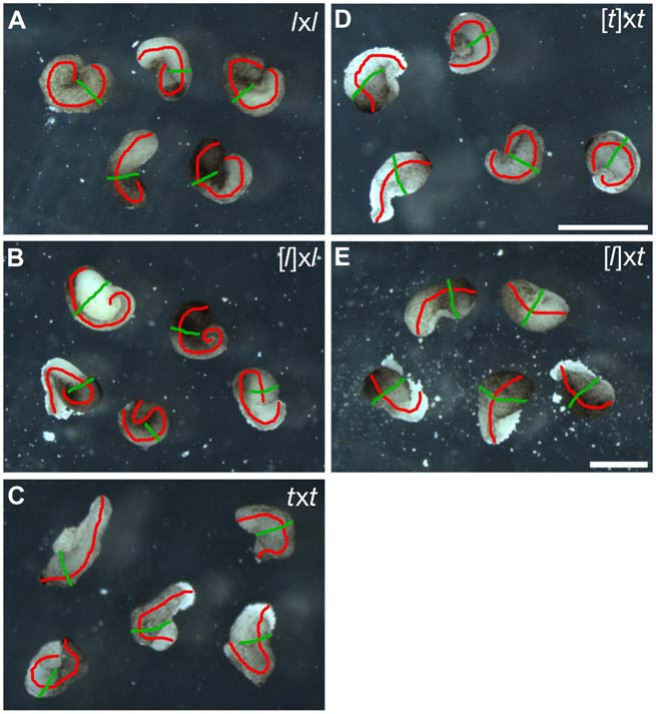
Reduced elongation of stage 10.5 dorso-marginal explants in cybrid embryos. Dorso-marginal explants were dissected from stage 10.5 (A) *l*x*l*, (B) [*l*]x*l*, (C) *t*x*t*, (D) [*t*]x*t*, and (E) [*l*]x*t* embryos and cultured overnight in 1× MBS. The axis of elongation (length) was highlighted in red and the width, in green. Five representative conjugates are shown for each experiment; see Table 2 for total numbers. Scale bars in (A, B, E) and (C, D): 0.5 mm.

To gain insights into the mechanisms responsible for the developmental faults of [*l*]x*t* cybrid embryos, we sought to understand the basis of their early gastrulation defect, namely the failure to close their blastopore and elongate their body axis (Figure 1, Video S2). Blastopore closure and body axis elongation are both highly dependent on efficient convergence and extension of the involuting marginal zone [49]. To test the efficiency of induction and convergence-extension movements in the gastrulating cybrid embryos, we compared the elongation of stage 10.5 dorso-marginal explants from these embryos to that of similar explants from control embryos. We adopted the following system to score the induction response [50]. If the explants are not induced, they remain spherical (no elongation). If induction and efficient convergence-extension occur, the explants elongate such that their length/width ratio becomes greater than two (well elongated). If the explants are induced but do not undergo efficient convergence-extension, they only partially elongate (stump). Over 70% of stage 10.5 dorso-marginal explants taken from control embryos (*l*x*l*, [*l*]x*l*, *t*x*t*, or [*t*]x*t*) underwent efficient convergence-extension, while the remaining also elongated, but to a lesser extent (Figure 4, Table 2). In contrast, few (14%) of the explants from [*l*]x*t* cybrid embryos underwent efficient convergence-extension, while most (67%) elongated to a lesser extent and some (19%) did not elongate (Figure 4, Table 2). Therefore, we conclude that the dorso-marginal region of [*l*]x*t* cybrid embryos is defective in induction response and convergence-extension during gastrulation, and this may be responsible, at least in part, for their incapacity to close their blastopore and properly elongate their body axis.

**Table 2:** Elongation of stage 10.5 dorso-marginal explants.

Dorso-Marginal Explants	Well Elongated (%)	Stump (%)	No Elongation (%)	Sample Size (Number of Experiments)
*l*x*l* a	23 (85)	4 (15)	0 (0)	27 (4)
[*l*]x*l* a	17 (74)	6 (26)	0 (0)	23 (3)
*t*x*t* a	12 (80)	3 (20)	0 (0)	15 (2)
[*t*]x*t* a	14 (82)	3 (18)	0 (0)	17 (2)
[*l*]x*t* b	3 (14)	14 (67)	4 (19)	21 (3)

a,b A relationship exists between explant kind and elongation (*p*<0.001; Chi-square analysis). Rows with different superscripts differ significantly (*p*<0.001) and rows with identical superscripts do not differ significantly (*p*>0.05) in pairwise Chi-square analyses.

### Poor Convergence-Extension in Gastrulating Cybrids Due to Deficient Induction Signalling and Response

**Figure 5:**
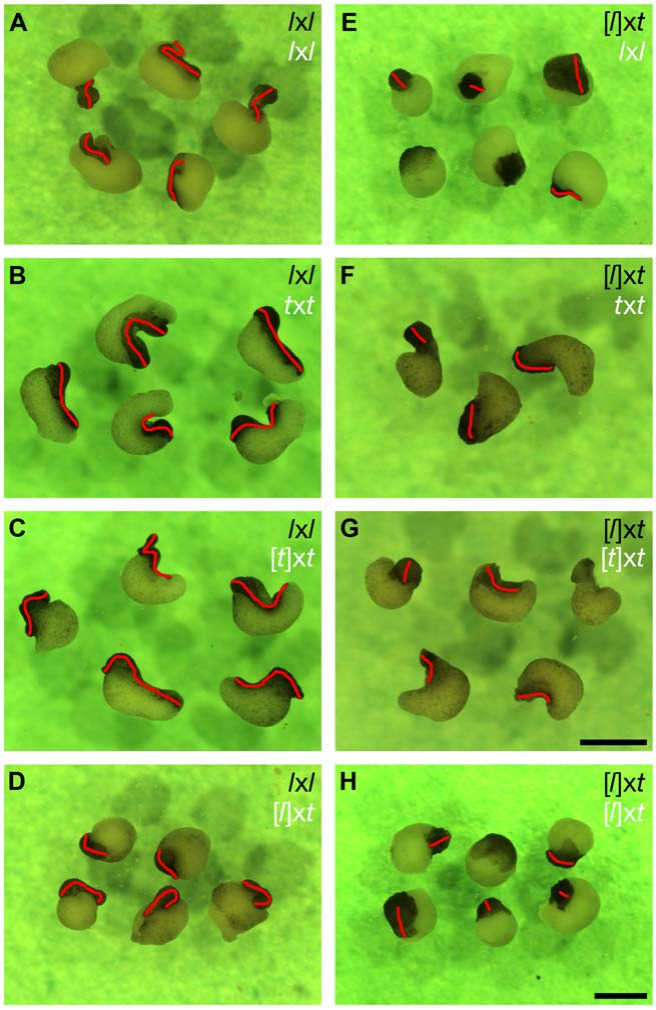
Elongation defects of cybrids result from deficient emission of, and response to, induction signals. Stage 8–9 animal caps (black inset) were dissected from (A–D) *l*x*l* or from (E–H) [*l*]x*t* cybrid embryos and conjugated to same-stage vegetal halves (white inset) of the following kinds of embryos (A, E) *l*x*l*, (B, F) *t*x*t*, (C, G) [*t*]x*t*, and (D, H) [*l*]x*t*. Animal-vegetal conjugations were cultured overnight in MBS. The axis of elongation, where present, was highlighted in red. 3–6 representative conjugates are shown for each experiment; see Table 3 for total numbers. Scale bars in (A, D, E, H) and (B, C, F, G): 1 mm.

Gastrulation movements and convergence-extension in *Xenopus* are driven by cells of the mesoderm, which arises at the equatorial region following the perception of a mesoderm-inducing signal that is generated by the vegetal hemisphere. If the cells of the animal hemisphere are not exposed to this signal, they remain ectodermal and do not elongate [51],[52]. Therefore, the reduced elongation response of the cells originating from the animal hemisphere in [*l*]x*t* cybrid embryos could in principle result either from deficient induction signal emission from the vegetal cells, or from a defective response of the animal cells to correct levels of induction signals, or both. To determine whether the vegetal hemisphere of the [*l*]x*t* cybrids secrete signals capable of inducing efficient convergence-extension in adjacent animal cells, we compared the elongation of naïve *l*x*l* animal caps (stages 8–9) that were conjugated to same-stage vegetal hemispheres of the following kinds: *l*x*l*, *t*x*t*, [*t*]x*t*, and [*l*]x*t*. Whereas the vegetal halves of control embryos (*l*x*l*, *t*x*t*, and [*t*]x*t*) were equally good at inducing *l*x*l* animal cap elongation, there was a marked reduction in the proportion of animal caps efficiently elongating following induction by the vegetal hemisphere of [*l*]x*t* cybrids (Figure 5, Table 3), suggesting that reduced emission of inductive signals by the vegetal half of the cybrid embryos may contribute to their convergence-extension defects. Nonetheless, a significant proportion (30%) of the *l*x*l* animal caps that were conjugated to [*l*]x*t* cybrid vegetal halves demonstrated efficient elongation, identical to the controls, suggesting that the vegetal cells of [*l*]x*t* cybrids can provide sufficient mesoderm-inducing signals to trigger efficient convergence-extension and elongation of animal cap cells, albeit in a reduced proportion of embryos (Figure 5, Table 3). It seems therefore unlikely that the only problem underlying the gastrulation defects, which occur in 100% of [*l*]x*t* cybrid embryos, is a deficient secretion of mesoderm-inducing signals by their vegetal hemisphere, although this may indeed contribute to the problem in many embryos.

**Table 3:** Animal cap elongation in different species combinations.

Animal Cap	Vegetal Cap	Well Elongated (%)	Stump (%)	No Elongation (%)	Sample Size (Number of Experiments)
*l*x*l*	*l*x*l* a	33 (75)	10 (23)	1 (2)	44 (6)
	*t*x*t* a	18 (86)	3 (14)	0 (0)	21 (4)
	[*t*]x*t* a	31 (89)	4 (11)	0 (0)	35 (5)
	[*l*]x*t* b	13 (30)	27 (63)	3 (7)	43 (7)
[*l*]x*t*	*l*x*l* c	6 (19)	21 (68)	4 (13)	31 (5)
	*t*x*t* c	6 (29)	15 (71)	0 (0)	21 (4)
	[*t*]x*t* c	4 (15)	19 (70)	4 (15)	27 (3)
	[*l*]x*t* c	2 (8)	15 (60)	8 (32)	25 (4)
[*t*]x*t*	*l*x*l* d	12 (52)	9 (39)	2 (9)	23 (4)

a,b A relationship exists between vegetal cap kind and elongation of *l*x*l* animal caps (*p*<0.001; Chi-square analysis). Rows with different superscripts differ significantly (*p*<0.001) and rows with identical superscripts do not differ significantly (*p*>0.05) in pairwise Chi-square analyses.

c No significant relationship exists between vegetal cap kind and elongation of [*l*]x*t* animal caps (*p*>0.05; Chi-square analysis).

d Row does not differ significantly (*p*>0.05) versus *l*x*l* animal cap conjugated to *l*x*l* vegetal cap, but differs significantly (*p* = 0.04) versus [*l*]x*t* animal cap conjugated to *l*x*l* vegetal cap in pairwise Chi-square analysis.

We thus investigated the possibility that the cells of the animal hemisphere in [*l*]x*t* cybrid embryos do not respond properly, even to normal levels of mesoderm-inducing signals. We compared the response of unspecified animal caps (stages 8–9) isolated from [*l*]x*t* cybrid embryos that were conjugated to diverse kinds of same-stage vegetal hemispheres (*l*x*l*, *t*x*t*, [*t*]x*t*, and [*l*]x*t*). Strikingly, elongation of these animal caps was only marginally (∼10%–20%) improved by their conjugation to any of the different non-cybrid vegetal halves tested (*l*x*l*, *t*x*t*, [*t*]x*t*) (Figure 5, Table 3). This suggests that the animal cap cells in the majority of the cybrid embryos do not undergo efficient convergence-extension, even if exposed to normal levels of mesoderm-inducing signals, coming from the vegetal hemispheres of either species' embryos. In contrast, the elongation of [*t*]x*t* animal caps in this assay was not significantly different from that of *l*x*l* (Table 3), confirming that the poor elongation of [*l*]x*t* animal caps is not solely the result of their ploidy. Therefore, the reduced elongation response of animal cap cells of [*l*]x*t* cybrid embryos results both from deficient induction signal emission from their vegetal hemisphere and from a defective response of their animal cells, even to a normal level of inductive signals.

### Induction Response and Convergence-Extension in Cybrids Are Partially Rescued by Increased Activin and *Xbra* Dosage

**Figure 6:**
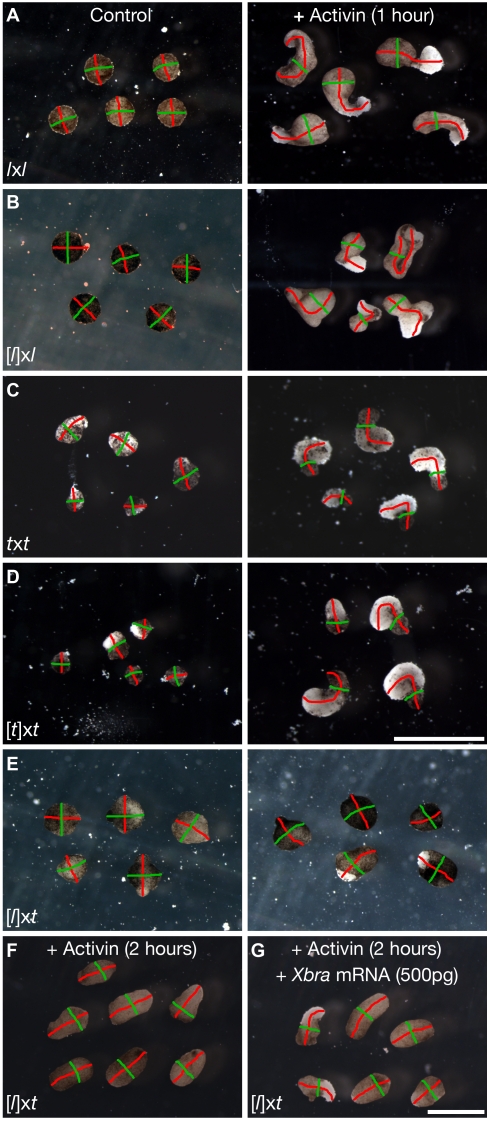
Activin-induced animal cap elongation is defective in cybrid embryos. Animal caps dissected from stage 8 (A) *l*x*l*, (B) [*l*]x*l*, (C) *t*x*t*, (D) [*t*]x*t*, and (E) [*l*]x*t* embryos were cultured either directly in MBS (left), or in MBS supplemented with 5 ng/ml Activin for 1 h, washed, and transferred to MBS overnight (right). (F–G) Induction and elongation response of [*l*]x*t* cybrid embryo animal caps are improved if (F) Activin exposure is doubled and improved further if (G) *Xbra* is also overexpressed. The axis of elongation (length) is highlighted in red and the width, in green. 4–7 representative explants are shown for each experiment; see Table 4 for total numbers. Scale bars in (A, B, E, F–G) and (C, D): 1 mm.

Mesoderm specification and animal cap elongation can be induced in vitro in a dish containing nanomolar concentrations of Activin A in a dose-dependent manner [51],[52]. In such an assay, animal caps isolated from *X. laevis* or *X. tropicalis* diploid embryos both have the same competence to respond to activin in terms of the induction of differentiation and gene expression [53]. We used this system to further test the induction and elongation efficiency of stage 8 animal cap cells isolated from [*l*]x*t* cybrid embryos. As expected, a significant proportion of the animal caps isolated from control embryos (*l*x*l*, [*l*]x*l*, *t*x*t*, and [*t*]x*t*) elongated well in response to activin in a dose-dependent manner (5 ng/ml for 20 or 60 min), although the elongation was generally less efficient in haploid embryos (Figure 6, Table 4). This was expected since axis elongation in haploid embryos is reduced compared to diploids (Figure 1; [34],[35]). A reduced proportion of naïve animal caps isolated from [*l*]x*t* cybrid embryos elongated in response to similar doses of activin in a dose-dependent manner, but strikingly they never underwent efficient convergence-extension (Figure 6, Table 4). This result confirms that the reduced convergence-extension in the cybrid embryos largely results from a deficient response of the animal cap cells, even to normal levels of mesoderm-inducing signals.

**Table 4:** Activin-induced animal cap elongation in different species combinations.

Animal Cap	Activin (5 ng/ml)¥	Well Elongated (%)	Stump (%)	No Elongation (%)	Sample Size (Number of Experiments)
*l*x*l*	—	0 (0)	0 (0)	46 (100)	46 (6)
	20′^a^	6 (40)	8 (53)	1 (7)	15 (2)
	60′^a^	32 (67)	13 (27)	3 (6)	48 (6)
	120′^a^	9 (32)	17 (61)	2 (7)	28 (3)
[*l*]x*l*	—	0 (0)	1 (3)	37 (97)	38 (6)
	20′^a^	7 (35)	9 (45)	4 (20)	20 (2)
	60′^a^	21 (50)	16 (38)	5 (12)	42 (6)
*t*x*t*	—	0 (0)	0 (0)	24 (100)	24 (4)
	20′^a^	11 (48)	8 (35)	4 (17)	23 (2)
	60′^a^	21 (72)	8 (28)	0 (0)	29 (4)
[*t*]x*t*	—	0 (0)	0 (0)	21 (100)	21 (4)
	20′^a^	3 (17)	7 (39)	8 (44)	18 (2)
	60′^a^	7 (27)	13 (50)	6 (23)	26 (4)
[*l*]x*t*	—	0 (0)	2 (4)	43 (96)	45 (6)
	20′^b^	0 (0)	3 (11)	24 (89)	27 (3)
	60′^a^^,^ c	0 (0)	45 (46)	52 (54)	97 (11)
	60′ (25 ng/ml)^a^^,^ d	1 (4)	19 (70)	7 (26)	27 (3)
	120′^a^^,^ e	3 (10)	23 (74)	5 (16)	31 (4)
	60′ (500 pg *Xbra* mRNA)^a^^,^ f	4 (9)	23 (49)	20 (43)	47 (5)
	120′ (500 pg *Xbra* mRNA)^a^^,^ e	11 (29)	20 (53)	7 (18)	38 (3)

¥ Stage 8 animal caps were treated with 5 ng/ml Activin A unless otherwise noted, for the indicated number of minutes. Where indicated, embryos were injected with 500 pg *Xbra* mRNA in the animal pole at stage 1. For each animal cap kind, a relationship exists between the Activin treatment and elongation. For both 20′ and 60′ Activin treatments, a relationship exists between animal cap kind and elongation (all *p* values<0.001; Chi-square analysis).

a Row differs significantly (*p*<0.001) in pairwise Chi-square analysis versus its respective no Activin control.

b,c Row differs significantly (b, *p*<0.005; c, *p*<0.001) in pairwise Chi-square analysis versus all other similarly treated animal caps.

d,e,f Row differs significantly (d, *p* = 0.009; e, *p*<0.001; f, *p* = 0.01) in pairwise Chi-square analysis versus 60′ Activin (5 ng/ml) [*l*]x*t* treated animal caps.

If the sensitivity to activin is compromised in [*l*]x*t* cybrid embryos, further increasing the activin induction treatment might be expected to rescue their defects in induction response and convergence-extension. Increasing the activin treatment, either by quintupling activin concentration or doubling the treatment time, indeed caused a higher proportion of the animal caps to elongate, and a few even underwent efficient convergence-extension (Figure 6, Table 4). These results indicate that the sensitivity to activin is compromised in [*l*]x*t* cybrid embryos. However, even if the induction treatment is increased to ensure the perception of induction signals (and an elongation response) in almost all embryos, the vast majority of these still do not undergo efficient convergence-extension (Figure 6, Table 4), suggesting that other incompatibilities manifest themselves by preventing efficient convergence-extension to occur during gastrulation.

We observed that Xbra protein concentration is markedly lower in all *X. laevis* egg-based embryos (*l*x*l*, [*l*]x*l*, [*l*]x*t*) compared to *X. tropicalis* egg-based embryos (*t*x*t*, [*t*]x*t*) (Figure 2B–C). Following the induction of mesodermal cells, one function of *Xbra* consists of suppressing migratory movements to instead promote convergence-extension [54]–[56]. One possibility is therefore that a lower (*X. laevis*–like) concentration of Xbra protein does not suppress cell migration enough to permit convergence-extension in cells with a *X. tropicalis* genome. To test this hypothesis, we overexpressed *Xbra* in [*l*]x*t* cybrid animal caps prior to activin treatment. As expected, such treatment did not affect the proportion of cybrid animal caps that responded to activin induction by undergoing some degree of elongation, while a few of them underwent efficient convergence-extension (Table 4). When combined with prolonged activin exposure, this treatment rescued convergence-extension in 29% of cybrid animal caps (Figure 6, Table 4). These results together suggest that the maternally regulated difference in Xbra protein concentration between the two species is partly responsible for the inefficient convergence-extension in gastrulating [*l*]x*t* cybrids, while reduced mesoderm-inducing signal emission and sensitivity also contributes to this phenotype.

**Figure 7:**
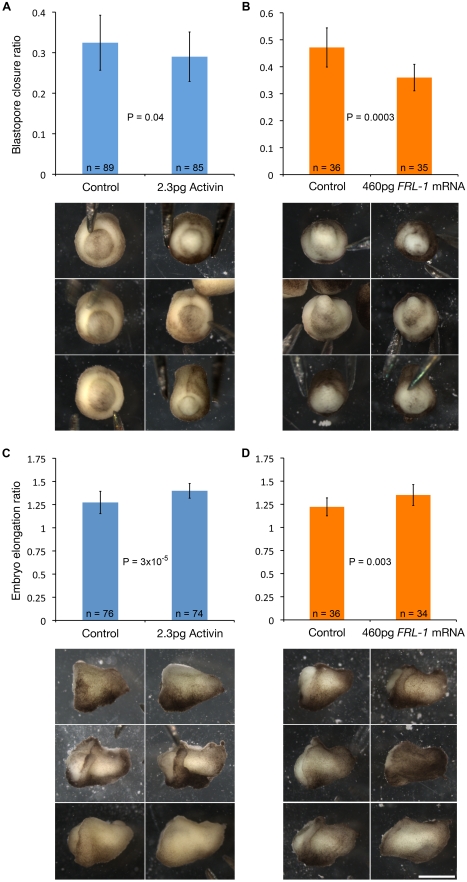
Increased induction signalling partially rescues convergence-extension in whole cybrid embryos. Injection of (A, C) human recombinant Activin A protein into the blastocoel of stage 8, or (B, D) FRL-1 mRNA in the animal half of one-cell [*l*]x*t* cybrid embryos, significantly improved (A, B) blastopore closure and (C, D) embryo elongation. Three representative H_2_O control (left) or Activin/*FRL-1*-treated (right) embryos are shown below each graph. The blastopore closure ratio (blastopore area/embryo area) was calculated from the respective diameters of the blastopore and embryo at 22 h post-fertilization. The embryo elongation ratio was obtained by dividing the embryo length by the greatest of the height or width at 32 or 46 h post-fertilization. Error bars represent the standard deviation between the means of 4 (Activin) or 3 (*FRL-1*) replicate experiments. *p* values were obtained using the one-tailed *t* test. n, sample size. Scale bar: 1 mm.

To validate these conclusions, we have attempted convergence-extension rescue in whole cybrid embryos using means expected to upregulate induction and/or *Xbra* signalling. One consisted in the injection of Activin A protein into the blastocoel of [*l*]x*t* cybrid blastulae, and the second in widely overexpressing *FRL-1*, an EGF-CFC family member that is a limiting co-factor in *nodal* signalling and mesoderm induction [57],[58]. These treatments both significantly improved blastopore closure and embryo elongation (Figure 7), two processes whose success is highly dependent on efficient convergence-extension. Widely overexpressing *Xbra* in whole cybrid embryos also improved elongation (*p* = 4×10^−7^), but it impaired blastopore closure and the resulting embryos were highly abnormal (unpublished data). These results further support the hypothesis that the nucleocytoplasmic incompatibilities that lead to inefficient convergence-extension in [*l*]x*t* cybrid embryos result from deficient induction signalling and response, and from inadequate Xbra protein concentration.

## Discussion

It is estimated that *X. laevis* and *X. tropicalis* diverged from a common ancestor approximately 50–65 million years (MY) ago [59]–[62]. In comparison, humans are separated from the common chimpanzee by only approximately 6 MY [30], while the extant placental mammal lineage evolved over approximately 135 MY [63],[64]. Considering previous iSCNT reports of EGA defects in many lethal cybrids [18],[24]–[27], it was somewhat surprising to observe normal activation of key embryonic genes in our amphibian cybrid. However, the evolutionary separation between the mammalian and amphibian combinations tested in these articles is more considerable, respectively, ranging from ∼65 to ∼100 MY, and ∼235 MY [61],[65]. Also, given that in *X. laevis* and *X. tropicalis* the expression patterns of transgenes generated with promoters from one species are generally maintained in the other species [66], and that there is a high level of amino acid identity (>98% overall) between the homologous proteins of each species [67], this result was not entirely unexpected. Moreover, because interspecies differences in transcription factor binding and gene expression are primarily directed by genetic sequence rather than cellular components [68], in our case *X. laevis* transcription factors are expected to bind to and promote transcription of *X. tropicalis* genes in a *X. tropicalis* specific manner. Our data also corroborate a study in a lethal loach–goldfish cybrid in which early embryonic expression of two genes (*ntl* and *gsc*) took place normally [23]. Since only a handful of genes were tested in both cases, it remains possible that the embryonic transcription of other genes is aberrant in these “EGA-successful” cybrid embryos, and genome-wide transcriptional analyses will be necessary to address this question. One such study recently reported that rhesus-bovine iSCNT embryos activated hundreds of genes at EGA to levels comparable with in vitro fertilized rhesus embryos [69].

Defects in rRNA synthesis and nucleologenesis (last step of EGA) have been noticed in two distantly related cybrids, including those generated by the iSCNT of *D. pictus* nuclei into *X. laevis* eggs [18],[26]. In both of these reports, however, mRNA synthesis (first step of EGA) was also substantially reduced, and thus it is conceivable that rRNA synthesis and nucleologenesis defects occurred as a secondary effect of the reduced mRNA synthesis rather than from an interspecies incompatibility. Alternatively, it is possible that the increased evolutionary distances between the combinations studied in these reports induced two independent incompatibilities leading to defects in both mRNA and rRNA synthesis. We did not observe any apparent nucleologenesis defects in our *Xenopus* cybrid. Also, since the terminal phenotype of anucleolate, rRNA-synthesis deficient, *X. laevis* mutant embryos [70],[71] is considerably less severe than that of *X. laevis* egg-based cybrids [14],[18], it seems rather unlikely that potential defects in nucleologenesis and rRNA synthesis could be responsible for, or even contribute to, their observed developmental defects. Cybrid lethality may therefore occur even if the egg cytoplasm is able to properly activate a foreign nucleus' genome, such that the last stage of EGA is completed. The incompatibility of [*l*]x*t* cybrids does not result from interference of the foreign nucleus on the maternally programmed early developmental processes because the presence of a *X. tropicalis* sperm nucleus does not impair (but improves) gynogenetic *X. laevis* haploid development. We therefore conclude that cybrid lethality may occur even if the donor nucleus does not interfere with the recipient cytoplasm-regulated development, while the latter competently “activates” the donor species' nucleus.

That we found no evidence of ATP deficiency or energy stress in [*l*]x*t* cybrid embryos was also somewhat unexpected, since it was shown that the combination of nuclei and mitochondria from cell lines of less distantly related mammalian species results in fatal defects in oxidative respiration [30],[31]. In fact, our analysis does not exclude the possibility that ATP/energy-related nucleo-mitochondrial incompatibilities exist between *X. laevis* and *X. tropicalis*. If they do exist, however, they are not manifested early enough to generate energy stress in time to be responsible for the initial developmental defects that are apparent in [*l*]x*t* cybrid embryos. Alternatively, it remains possible that other, ATP/energy-unrelated, nucleo-mitochondrial incompatibilities could contribute to the gastrulation defects of the cybrids.

We provide evidence that gastrulating cybrid embryos cannot execute efficient convergence-extension movements because of reduced levels of mesoderm-inducing signal emission by the vegetal pole of some of these, but also largely due to a defective elongation response of animal cap cells, in most if not all embryos. Exactly why this is taking place in the cybrids remains unclear, yet elongation of the animal cap cells of the cybrids can be partially rescued in explants and whole embryos by increasing exposure to a mesoderm-inducing signal (Activin). One could hypothesize that the difference in egg sizes between the two species could affect induction signalling, or gastrulation movements. This, however, seems unlikely since our experiments clearly showed that the elongation defects of [*l*]x*t* cybrids occurred even in explants in vitro. Furthermore, the development of reciprocal inter-subspecies cybrids from *X. laevis laevis* and *X. laevis victorianus* was completely normal, despite over a 3-fold difference in the volume of their eggs [72].

We further show that the concentration of a key embryonic protein (Xbra) is different in the embryos of the two *Xenopus* species, while this concentration appears to be under cytoplasmic (maternal) control in cybrids. Since reduced *Xbra* activity inhibits convergence-extension in *X. laevis* [54]–[56], and since *Xbra* overexpression partially rescues this defect in cybrids, it suggests that differences in key protein concentrations between species constitutes a form of nucleocytoplasmic incompatibility that contributes to developmental defects and lethality in cybrids. This phenomenon is likely not restricted to Xbra, since β-actin concentration is also markedly increased in *X. tropicalis* eggs and embryos compared to *X. laevis* (unpublished data), while it was recently shown that different concentrations of Importin α and Ntf2 are responsible for the divergent nuclear sizes between these two species [67]. The mechanisms regulating protein concentration are largely unexplored, but from our analysis it appears that embryonic protein concentrations are under cytoplasmic (maternal) control, and not merely a reflection of mRNA concentrations, while inappropriate concentrations of key proteins in [*l*]x*t* cybrids, such as Xbra and likely others, may underlie their observed developmental defects and lethality. Quantitative proteomics analysis in [*l*]x*t* and other kinds of cybrids should reveal the magnitude of this mechanism.

Finally, our (and others [73]) results demonstrate that it is possible to correct nucleocytoplasmic incompatibilities of cybrids by appropriate treatments. If nuclear transfer remains the most effective method to derive Embryonic Stem (ES) cells from adult tissues [74], iSCNT using the oocytes of a more available species followed by the injection of these iSCNT-derived ES cells into a host blastocyst of the oocyte species could constitute an optimal route towards the generation of immuno-compatible organs of the donor species within the developing body of the recipient species [75]. A better understanding of the nucleocytoplasmic incompatibilities causing cybrid lethality may enable their correction and render such technology possible.

## Materials and Methods

### *Xenopus* Eggs and Embryos

*Xenopus laevis* and *Xenopus tropicalis* adults were purchased from Nasco and maintained in our laboratory in separate systems, respectively operating at 18°C and 26°C. Eggs were collected dry onto dishes by gently massaging the frog's flanks [76], since this was found to increase cross-fertilization efficiency. For nuclear inactivation, eggs were individually placed in a plastic dish with their animal pole facing up and submitted to UV irradiation for 25–30 s as previously described [32]. Fertilization or cross-fertilization was done by gently mixing the eggs with a crushed testis solution [76]. For sperm nuclear inactivation, a small clump-free volume taken from a crushed testis solution was spread on a glass slide to form a thin layer and exposed to UV irradiation for 20 s, using the same apparatus as for egg nuclear inactivation [32]. Such treatment resulted in 100% sperm DNA inactivation as determined by the characteristic haploid phenotype of the resulting embryos and examination of their nucleolar numbers. The optimal temperature range at which *X. laevis* and *X. tropicalis* are raised is different, but both species can develop normally at comparable rates at an intermediate temperature of 23°C [66],[77], and thus we have performed all of our experiments at this temperature. All embryos were maintained in 1/10 MMR unless otherwise mentioned, and staged according to the normal table of *X. laevis* development [78].

### mRNA and Activin Injection

Cross-fertilized enucleated *X. laevis* eggs were de-jellied using a 2% L-Cysteine (pH 8) solution, placed in a 6% Ficoll (type 400), 4/10 MMR solution [79], and injected with distilled H_2_O (_d_H_2_O), 460pg *FRL-1(UTR-)* [58], or 500 pg *Xbra* [80] in vitro synthesized capped mRNA in their animal half at the one-cell stage, or with _d_H_2_O or 2.3 pg recombinant human Activin A protein in their blastocoel at stage 8, using a Drummond micro-injector.

### Immunostaining and Karyotype Analysis

For karyotype analysis, embryos between stages 20 and 34 were cut open and immersed in _d_H_2_O for 20 min, fixed in 60% acetic acid for 5 min, and squashed onto a polysine microscope slide by pressing a coverslip down against it firmly. Slides were put on dry ice for 5 min, and then the coverslip was flicked off. The slide was then immersed in 20 µM Hoechst for 5 min, quickly drained, and 5 µl of vectashield mounting medium was added before covering with a new coverslip and sealing with nail polish. For immunostaining, embryos were harvested after 72 h of development and the dorsal (yolk-free) region was squashed on a polysine slide followed by freeze-cracking as above. They were then fixed in 4% paraformaldehyde for 30 min and blocked in 1% BSA for 1 h. Monoclonal mouse anti-fibrillarin (AbCam; 1∶400 dilution) and monoclonal Alexa 488-coupled anti-mouse (Invitrogen; 1∶250 dilution) secondary antibodies were used. DAPI (1 µg/ml) was used as a counterstain.

### ATP Measurements

Eggs/embryos were de-jellied and collected at various time points after fertilization. Five eggs/embryos were transferred to an eppendorf tube, rinsed once in _d_H_2_O, and resupended in 250 µl of _d_H_2_O. Eggs/embryos were then homogenized by passing through a 26G syringe several times. The resulting suspension was chilled on ice and spun at 13,000 rpm for 5 min. 50 µl was then taken from the supernatant and transferred to a new tube where it was homogenized by pipetting. 25 µl of this was diluted 5-fold in _d_H_2_O, and kept on ice until assayed. ATP was then measured using an ATP Bioluminescent Assay kit (Sigma) (at 1/500 dilution of the ATP assay mix) according to the manufacturer's recommendation and a Glomax luminometer.

### Western Blotting

Western blotting was performed essentially as previously described [38]. One *X. laevis* egg/embryo or 4.75 *X. tropicalis* eggs/embryos were loaded per well. (This volume ratio was estimated based on the approximate diameter ratio (0.59) of the eggs of *X. tropicalis/X. laevis* utilized in our experiments.) Rabbit monoclonal anti-phospho-AMPK (Cell Signaling; 1∶1,000 dilution), mouse monoclonal anti-Ran (BD Biosciences; 1∶2,000 dilution), rabbit polyclonal anti-Xbra (raised against *X. laevis* Xbra) [80] primary, and Alexa Fluor 680-conjugated goat anti-rabbit (Invitrogen; 1∶20,000 dilution) and 800CW-conjugated goat anti-mouse (LI-COR; 1∶20,000 dilution) secondary antibodies were used. Fluorescence was detected using the Odyssey detection system from LI-COR.

### Real-Time RT-PCR

Total RNA was prepared from single egg/embryo (*X. laevis*) or pools of three (*X. tropicalis*) using the RNeasy kit (Qiagen) and eluted in 20 µl. Total RNA concentration in each sample was determined by optical density at 260 nm. 10 µl of total RNA was used for real-time RT-PCR as previously described [81] using SYBR Green. Primers were as follows (5′→3′): *Vegt* F: catcgctacaagcccaggtt, R: caatccccatggagaattgtaca, *Xbra* F: gaatgtgctggcaaagggtaa, R: ttccgttttcctgcatctttaaa, *Chordin* F: gctcagcaggtcacgcatgg, R: gttaggtatgtgcacttgtc, *GATA4* F: gcttaaaactctcgccacaga, R: tgctttaagctaagaccaggttg, *Mixer* F: cagcagaggttcctgatgc, R: taagaggcaggaattccatggt. *Vegt* primers amplified both *X. laevis* and *X. tropicalis* sequences. Relative mRNA quantities were normalized for total RNA input for each sample before comparison.

### Elongation Assays

Explants were isolated in 1× MBS using fine forceps and a scalpel blade. Dorso-marginal zones were collected from stage 10.5 embryos essentially as previously described [82]. Animal-vegetal conjugates were constructed using stage 8–9 embryos as previously described [76]. For the activin conditioning assay, animal caps were collected from stage 8 embryos, incubated in 1× MBS with or without human recombinant activin A (R&D Systems) at various concentrations for various durations and washed. In every case, animal cap elongation was scored as previously described [50], by the determination of their length∶width ratio after overnight incubation at 23°C in 1× MBS supplemented with antibiotics in 2% agarose-lined dishes, after verifying that sibling embryos had reached stage 19.

## Supporting Information

Figure S1 Characterization of the hybrids formed by the cross-fertilization of *X. laevis* eggs with *X. tropicalis* sperm. (**A**-**C**) *l*x*t* embryos are *bona fide* hybrids and synthesize proteins from the *X. tropicalis* genome. Stage 40 tadpoles of the following kinds are shown: (A) *l*x*l*, (B) *albino* (*a*) *l*x*l* (*al*x*al*) and (C) *al*x*t*. 22/22 stage 40 *al*x*t* tadpoles had a wild-type pigmentation pattern, indicating expression from the *X. tropicalis albino* gene. (**D**-**E**) Karyotype analysis revealed the expected chromosomal content in cells of *l*x*t* hybrids based on the respective haploid complement of each species (*X. tropicalis*: 10; *X. laevis*: 18). Hoechst-stained metaphase nuclei spreads from (D) [*t*]x*t* (10 chromosomes) and (E) *l*x*t* hybrid (28 chromosomes) stage 32 tadpoles are shown. Arrowheads point at the *X. tropicalis* marker chromosome 10 which is distinguishably smaller than all the other chromosomes present in each species. Individual chromosomes were manually highlighted in red. (**F**-**H**) *l*x*t* hybrids can metamorphose and develop into mature adults that have an intermediate phenotype relative to the two parental species. Adult (F) *l*x*l*, (G) *l*x*t*, and (H) *t*x*t* males are shown. Scale bars in (A-C): 1 mm; (D-E): 2 mm. Diameter of the coin present in the background of (F-G): 2 cm; (G-H) are shown at the same magnification.
(TIF)

Figure S2 Nucleologenesis appears normal in cybrid embryos. (**A**-**F**) No obvious difference was noticeable under differential interference contrast microscopy in the appearance of the nucleoli (darker spots) present in the nuclei of (A) *l*x*l*, (B) [*l*]x*l*, (C) *t*x*t*, (D) [*t*]x*t*, (E) *l*x*t*, and (F) [*l*]x*t*. (**G**) Quantification of the number of nucleoli/nucleus revealed no significant difference between *l*x*l* and *t*x*t*, or between [*l*]x*l*, [*t*]x*t*, and [*l*]x*t* embryos (all P values > 0.05). The percentage of nuclei having two nucleoli in *l*x*t* hybrids is significantly reduced compared to *l*x*l* and *t*x*t* diploids (P values < 0.001). 8 to 12 embryos from 2 to 3 different crosses were analysed for each kind of embryos. The one-tailed t-test with unequal variance was used for statistical analysis. Actively dividing cells were excluded from this analysis. (**H**-**J**) Nucleolar integrity in [*l*]x*t* cybrids was confirmed by the correct distribution of Fibrillarin (green), detected with monoclonal anti-fibrillarin antibodies. DAPI (blue) was used to visualize DNA. Scale. bars in (A-F) and (H-J): 10 mm.
(TIF)

Text S1 Characterization of *l*x*t* hybrid development.
(DOC)

Video S1 Early cleavages and timing of gastrulation are unaffected in cybrid embryos. Animal view of the diverse kinds of *X. laevis* egg-based embryos reveals that haploid [*l*]x*l* (middle) and [*l*]x*t* cybrid (bottom) embryos cleave and begin gastrulation synchroneously, about 50 minutes after diploid *l*x*l* (top) embryos. A star was added to the right of embryos at the onset of gastrulation (stage 10), when embryo-wide cellular movements begin. Images were captured every 4 minutes at ∼26°C, and displayed at a rate of 10 images per second.
(MOV)

Video S2 Gastrulation defects in cybrid embryos. Vegetal view of the diverse kinds of *X. laevis* egg-based embryos reveals that haploid [*l*]x*l* (middle) and [*l*]x*t* cybrid (bottom) embryos begin gastrulation synchroneously, about 50 minutes after diploid *l*x*l* (top) embryos. [*l*]x*t* cybrid embryos however consistently fail to fully close their blastopore and form abnormal neurulae. Images were captured every 4 minutes at ∼25°C, and displayed at a rate of 10 images per second.
(MOV)

## Abbreviations

AMPK: AMP-activated protein kinase
cybrid: nucleocytoplasmic hybrid
EGA: embryonic genome activation
ES: embryonic stem
iSCNT: interspecies somatic cell nuclear transfer
MY: million years
UV: ultraviolet
