# Supplementary figures and images for: Deficient Induction Response in a Xenopus Nucleocytoplasmic Hybrid
Source: PLoS Biol. 2011 Nov 15;9(11):e1001197. doi: 10.1371/journal.pbio.1001197 (PMC3217020; doi:10.1371/journal.pbio.1001197)

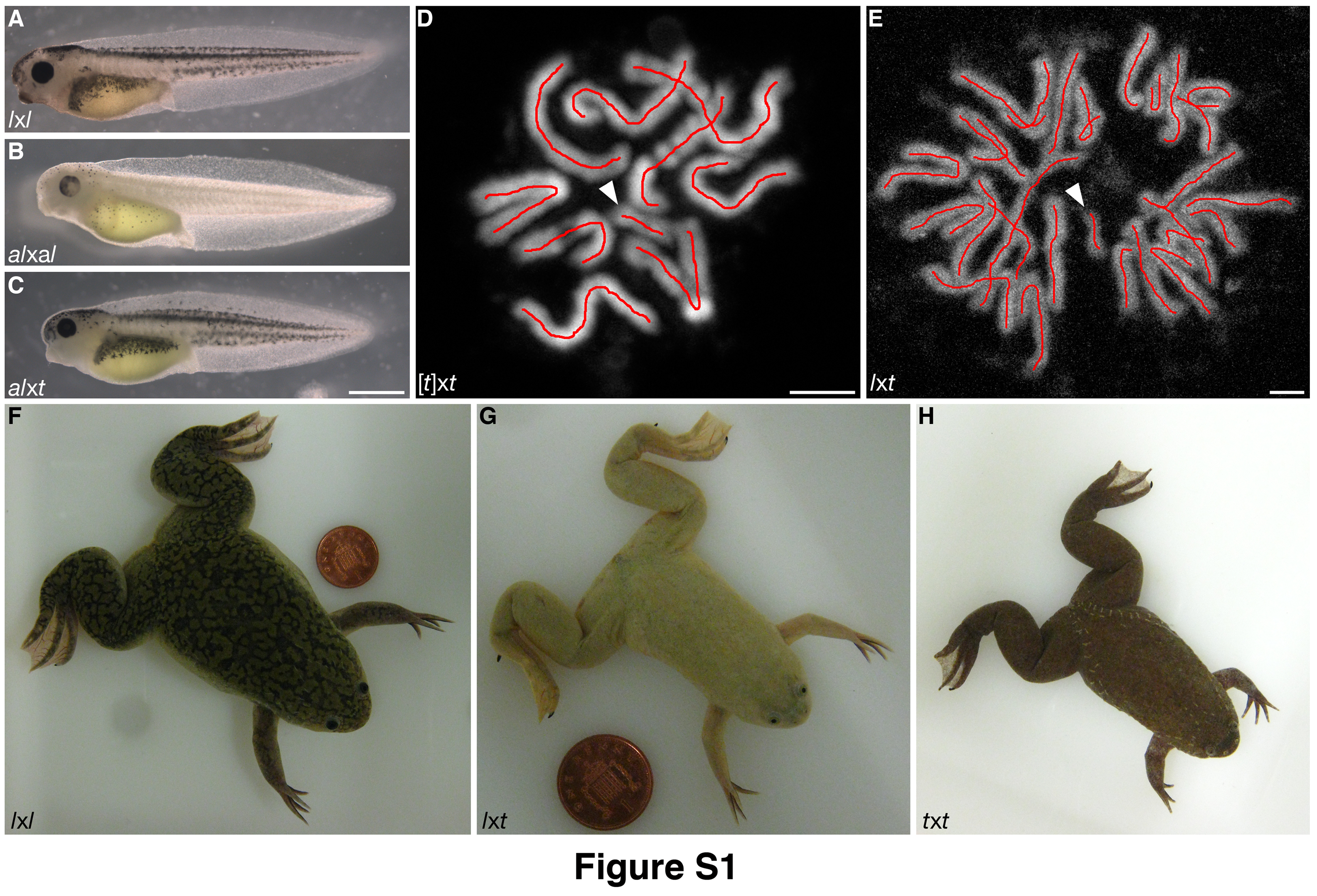

Supplement: Figure S1 — Characterization of the hybrids formed by the cross-fertilization of X. laevis eggs with X. tropicalis sperm. (A-C) lxt embryos are bona fide hybrids and synthesize proteins from the X. tropicalis genome. Stage 40 tadpoles of the following kinds are shown: (A) lxl, (B) albino (a) lxl (alxal) and (C) alxt. 22/22 stage 40 alxt tadpoles had a wild-type pigmentation pattern, indicating expression from the X. tropicalis albino gene. (D-E) Karyotype analysis revealed the expected chromosomal content in cells of lxt hybrids based on the respective haploid complement of each species (X. tropicalis: 10; X. laevis: 18). Hoechst-stained metaphase nuclei spreads from (D) [t]xt (10 chromosomes) and (E) lxt hybrid (28 chromosomes) stage 32 tadpoles are shown. Arrowheads point at the X. tropicalis marker chromosome 10 which is distinguishably smaller than all the other chromosomes present in each species. Individual chromosomes were manually highlighted in red. (F-H) lxt hybrids can metamorphose and develop into mature adults that have an intermediate phenotype relative to the two parental species. Adult (F) lxl, (G) lxt, and (H) txt males are shown. Scale bars in (A-C): 1 mm; (D-E): 2 mm. Diameter of the coin present in the background of (F-G): 2 cm; (G-H) are shown at the same magnification. (TIF) [file pbio.1001197.s001.tif]

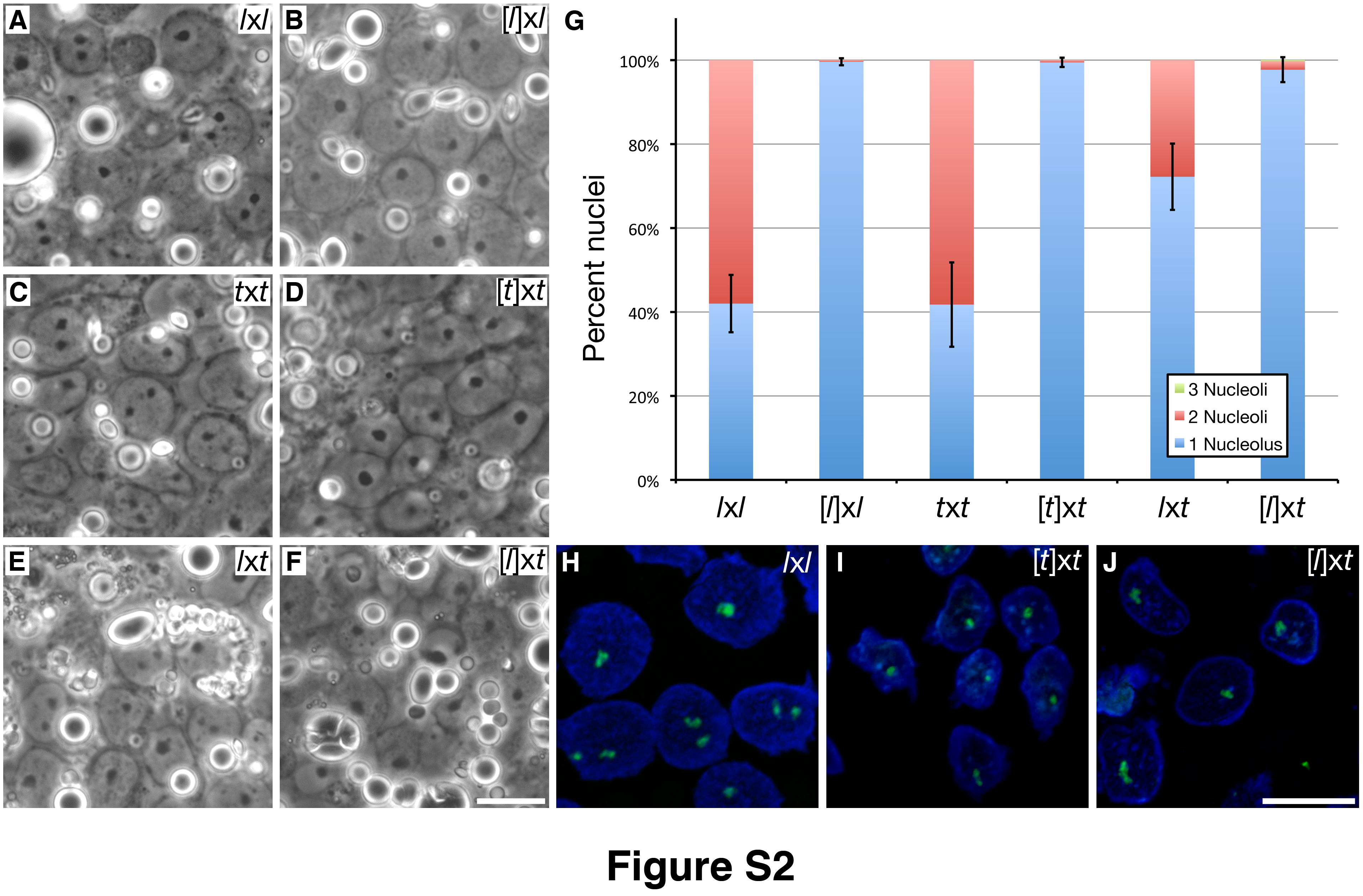

Supplement: Figure S2 — Nucleologenesis appears normal in cybrid embryos. (A-F) No obvious difference was noticeable under differential interference contrast microscopy in the appearance of the nucleoli (darker spots) present in the nuclei of (A) lxl, (B) [l]xl, (C) txt, (D) [t]xt, (E) lxt, and (F) [l]xt. (G) Quantification of the number of nucleoli/nucleus revealed no significant difference between lxl and txt, or between [l]xl, [t]xt, and [l]xt embryos (all P values > 0.05). The percentage of nuclei having two nucleoli in lxt hybrids is significantly reduced compared to lxl and txt diploids (P values < 0.001). 8 to 12 embryos from 2 to 3 different crosses were analysed for each kind of embryos. The one-tailed t-test with unequal variance was used for statistical analysis. Actively dividing cells were excluded from this analysis. (H-J) Nucleolar integrity in [l]xt cybrids was confirmed by the correct distribution of Fibrillarin (green), detected with monoclonal anti-fibrillarin antibodies. DAPI (blue) was used to visualize DNA. Scale. bars in (A-F) and (H-J): 10 mm. (TIF) [file pbio.1001197.s002.tif]
