# Supplementary material for: Deficient Induction Response in a Xenopus Nucleocytoplasmic Hybrid
Source: PLoS Biol. 2011 Nov 15;9(11):e1001197. doi: 10.1371/journal.pbio.1001197 (PMC3217020; doi:10.1371/journal.pbio.1001197)
Supplement: Text S1 — Characterization of lxt hybrid development. (DOC) [file pbio.1001197.s003.doc]

**Supporting Information**

**Text S1. Characterization of *l*x*t* hybrid development.** To verify that *l*x*t* embryos are *bona fide* hybrids and express genes from the *X. tropicalis* genome, we cross-fertilized *albino* *X. laevis* eggs with wild-type *X. tropicalis* sperm (*_albino_l*x*t*). Pigmentation was completely rescued in 100% of these hybrid embryos by stage 40 (Figure S1A-C), confirming the presence of, and expression from, the *X. tropicalis* genome. Cells in the hybrid embryos had 28 identifiable chromosomes in stage 32 metaphase spreads, corresponding to the sum of the haploid content of both species: 18 in *X. laevis* and 10 in *X. tropicalis* (Figure S1D-E), confirming the presence of one copy of each genome in the *l*x*t* hybrids.

Survival was however highly reduced in *l*x*t* hybrid embryos beyond stage 40 and up to the completion of metamorphosis; the large majority of these developed a range of defects including a kinked axis/tail, aberrant swimming and feeding positions, oedema, non-functional appendages, and died at various stages (data not shown). Out of over 1000 cross-fertilized embryos from 5 different male/female combinations, only two such hybrids successfully completed metamorphosis and so far (after approximately 1 year) only one has grown into a mature adult male as judged by its size, the appearance of dark patches (nuptial pads) on the ventral side of its forelimbs at maturity, and its mating behaviour. Its size and phenotype are intermediate between *X. laevis* and *X. tropicalis* (Figure S1F-H). Natural mating trials were unsuccessful with either *X. laevis* or *X. tropicalis* despite establishing amplexus with the females of both species, suggesting that *l*x*t* males are sterile (data not shown; O. Bronchain, *personal communication*).
